# The adaptive transition of glioblastoma stem cells and its implications on treatments

**DOI:** 10.1038/s41392-021-00491-w

**Published:** 2021-03-23

**Authors:** Zeyu Wang, Hao Zhang, Shengchao Xu, Zhixiong Liu, Quan Cheng

**Affiliations:** 1 Department of Neurosurgery, Xiangya Hospital, Central South University, Changsha, P.R. China; 2 National Clinical Research Center for Geriatric Disorders, Changsha, P.R. China; 3 Department of Clinical Pharmacology, Xiangya Hospital, Central South University, Changsha, P.R. China

**Keywords:** CNS cancer, Cancer stem cells

## Abstract

Glioblastoma is the most malignant tumor occurring in the human central nervous system with overall median survival time <14.6 months. Current treatments such as chemotherapy and radiotherapy cannot reach an optimal remission since tumor resistance to therapy remains a challenge. Glioblastoma stem cells are considered to be responsible for tumor resistance in treating glioblastoma. Previous studies reported two subtypes, proneural and mesenchymal, of glioblastoma stem cells manifesting different sensitivity to radiotherapy or chemotherapy. Mesenchymal glioblastoma stem cells, as well as tumor cells generate from which, showed resistance to radiochemotherapies. Besides, two metabolic patterns, glutamine or glucose dependent, of mesenchymal glioblastoma stem cells also manifested different sensitivity to radiochemotherapies. Glutamine dependent mesenchymal glioblastoma stem cells are more sensitive to radiotherapy than glucose-dependent ones. Therefore, the transition between proneural and mesenchymal subtypes, or between glutamine-dependent and glucose-dependent, might lead to tumor resistance to radiochemotherapies. Moreover, neural stem cells were also hypothesized to participate in glioblastoma stem cells mediated tumor resistance to radiochemotherapies. In this review, we summarized the basic characteristics, adaptive transition and implications of glioblastoma stem cells in glioblastoma therapy.

## Background

Glioblastoma (GBM), classified as grade IV glioma, is a highly aggressive and heterogeneous tumor in the central nervous system. Standard treatments of GBM include maximal surgical resection and following radiochemotherapies, which is also known as the STUPP protocol.^1^ Nevertheless, the average overall survival time is still <14.6 months for newly diagnosed GBM patients and 6.9 months for recurrence GBM patients.^2^ Novel treatments such as anti-angiogenic therapy,^3^ immunotherapy^4,5^ and tumor-treating electric fields^6^ were proposed recently but their efficacies were still unsatisfied. GBM is categorized into four subtypes based on their molecular characteristics: proneural, neural, mesenchymal and classical gliomas,^7–9^ and patients’ prognoses varied among those subtypes. In primary GBM, the mesenchymal GBM is the most aggressive type while the proneural GBM is associated with a relatively better overall survival compared to other subtypes. Moreover, mostly primary GBM experiences the subtype switch at relapse, in which mesenchymal GBM is the most stable subtype.^10^ Therefore, this GBM subtype switch is reckoned as an adaptive transition considered as an underlying mechanism of tumor resistance to radiochemotherapies.^11–13^

**Fig. 1:**
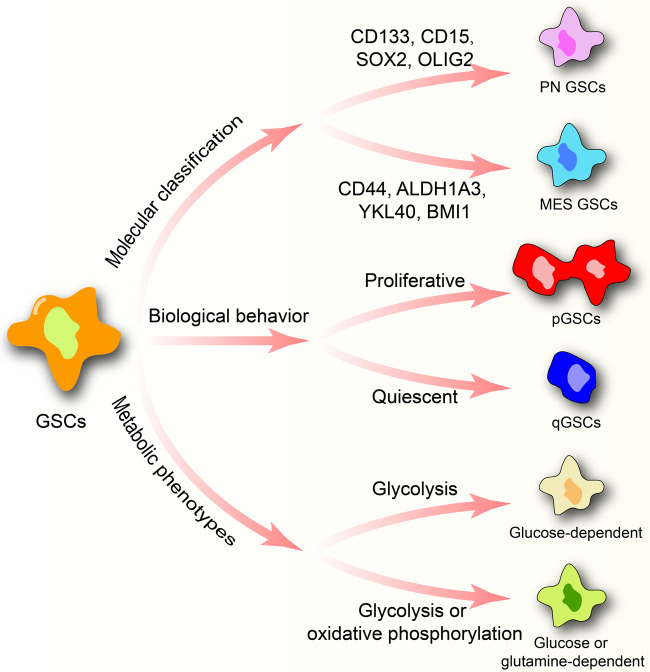
GSCs classification based on molecular signatures, metabolic phenotypes and biological behavior. The molecular classification includes PN GSCs and MES GSCs. Two metabolic phenotypes of GSCs are identified. The former mostly dependent on glycolysis. The latter metabolizes glutamine to supply OXPHOS but it can switch to glycolysis when glutamine is insufficient. According to biological behavior, GSCs can be grouped into proliferative GSCs and quiescent GSCs

Cancer stem cells were first isolated from acute myeloid leukemia by Bonnet and Dick in 1997,^14^ which is defined as a cluster of undifferentiated cells with the ability of self-renewal and tumor initiation. Glioblastoma stem cells (GSCs) were isolated from GBM with the ability to develop GBM in the transplanted mouse.^15,16^ Several biomarkers were identified to distinguish GSCs from non-tumorigenic stromal cells including CD56^+^, SOX2^+^, SOX9^+^, CD133^+^, CD15^+^, CD248^−^, CD105^−^, αSMA^−^ .^17^ Besides, GSCs were classified into different groups based on gene signatures,^18–24^ metabolic patterns^25,26^ and biological behaviors^25,27^ (Fig. 1). Those classifications were not isolated but were also closely connected. For example, GSCs are classified as proneural GSCs (PN GSCs) or mesenchymal GSCs (MES GSCs) based on gene signatures; in metabolic patterns, MES GSCs switches between glycolysis and oxidative phosphorylation (OXPHOS) whereas PN GSCs mainly dependent on glycolysis; PN GSCs and MES GSCs were characterized with potent proliferative and invasive abilities, respectively. The biological behavior classification, defining proliferative GSCs (pGSCs) and quiescent GSCs (qGSCs), is associate with neural stem cells (NSCs) but have no relationship with the molecular or metabolic classification.

Moreover, those classifications are also linked with tumor resistance to radiotherapy or chemotherapy. For instance, MES GSCs and qGSCs manifest relatively higher resistance to radiotherapy compared to PN GSCs and pGSCs, respectively.^28,29^ Glutamine-dependent MES GSCs show resistance to radiochemotherapies.^26,30,31^ Besides, PN GSCs can be induced to transform into MES GSCs by treating with temozolomide.^32^ Similar phenomenon was also noticed between pGSCs and qGSCs.^33^ Given that, we summarize the characteristics of each classification of GSCs, explore their internal relationships and investigate their association with tumor resistance to radiochemotherapies. Consequently, the phenomenon that therapy sensitive GSCs switch to therapy-resistant GSCs is summarized with the term, ‘the adaptive transition of GSCs’. The role of NSCs and niche acts during GSCs adaptive transition are also discussed. Finally, the implication of GSCs adaptive transition to clinical treatment is investigated to provide novel potential strategies for future GBM treatment.

## GSCs isolation

Currently, there are various methods to isolate GSCs including sphere-formation assay, side population assay, label-retention assay and flow cytometry.^34,35^ To verify the ability of self-renewal and tumor initiation, sphere-formation assay and GSCs allogeneic transplantation are required after isolation.^36^ Therefore, a regular protocol consists of isolation and verification. In order to obtain sufficient GSCs for verification, sphere-formation assay and flow cytometry assay are considered as qualified methods.^37^ In addition to those classical culture systems, 3D organoids system for GSCs was also proposed in recent years.^38–40^ Stem-like cells were also noticed during culturing GBM cells as 3D organoids in vitro. Moreover, high proliferative SOX2 positive GSCs enriched at the periphery of the organoids; while rare SOX2 positive GSCs were noticed at its hypoxic core. GSCs at the core of organoids exhibited worse proliferative ability by comparing with GSCs at the surface.^39^ Compared with the traditional sphere-forming assay, 3D organoids system highlighted the interaction between tumor cells and extracellular matrix (ECM) components.

In addition, proper culture medium selection is also critical to stem cells enrichment and the expression of surface markers on GSCs. For instance, serum-free medium can assist in isolating GSCs from tumor tissue and maintaining GSCs stemness.^41,42^ Cytokines like FGF has been proved with the ability of affecting surface marker of stem cells. Adding FGF into cell culturing medium affects the expression of Nestin and CD133, which are star biomarker of stem cells, and maintain the characteristics of GSCs.^43,44^ The Wnt signaling pathway can cross talk with FGF to influence cell surface marker expression, including CD133, CD44.^45^ Considering cell surface marker is critical to GSCs isolation and identification, we listed common hallmarks of GSCs along with their association with PN or MES GSCs. (Table 1).

**Table 1:** Common hallmarks in GSCs isolation

Hallmarks	Functions	Subtypes	Reference
CD133	Cell cycle and tumor cell proliferation.	PN GSCs	^46^
CD15	Cell proliferation, self-renewal, and multilineage differentiation.	PN GSCs	^51^
ITGA6	Cell proliferation and adhesion.	PN GSCs	^56,57^
A2B5	Cell proliferation, migration, clonogenicity, and tumorigenesis.	PN GSCs	^53^
CD44	Cell invasion	MES GSCs	^19,55^
ALDH1	Tumorigenesis, PMT transition, resistance to temozolomide, cell invasion, cell proliferation, glycolysis	MES GSCs	^58^
Nestin	A class VI intermediate filament protein	Not suitable	^105^
CD36	A scavenger receptor, GSCs self-renewal and proliferation.	Not suitable	^60^
CD9	Cell proliferation.	Not suitable	^61–63^
IL6R	Tumor growth	Not suitable	^64,65^
CXCR4	Cell proliferation, self-renewal.	Not suitable	^66,67^

CD133, also called prominin-1, is the most common hallmark be applied to GSCs isolation.^46^ CD133 is considered as a hallmark of PN GSCs while CD133 negative GSCs are considered as MES GSCs.^47^ A previous study reported that CD133 is associated with tumor angiogenesis, cell proliferation while CD133 negative GSCs lack the ability of self-renewal and forming sphere in vitro.^48^ Nevertheless, CD133 negative GSCs can form tumor in vivo and CD133 positive GSCs can be isolated from it.^47^ Therefore, isolation by targeting CD133 can obtain GSCs but may not be able to pure MES GSCs.

Similar to CD133, CD15 (known as SSEA-1 or Lex) can be applied as a target in GSCs isolation. The expression of CD133 in CD15 positive GSCs is deceased during passage in vitro while the expression of CD15 remains stable. Notably, CD15 positive CD133 negative cells still maintain the characteristic of GSCs in vitro.^49^ However, no significant difference of phenotypic and genomic characteristics is observed between CD15 positive GSCs and CD15 negative GSCs, which both can develop a CD15 positive/negative mixed tumor in vivo.^50^ Therefore, some researches adopted CD15 and CD133 as isolation hallmarks simultaneously to reduce omission.^51,52^

A2B5 is recognized as an isolation hallmark of GSCs.^53^ The ability of tumor initiation of A2B5 positive CD133 negative GSCs is stronger than A2B5 negative CD133 negative GSCs.^54^ A study reported that A2B5 negative GSCs failed to form sphere in vitro or tumor in vivo.^53^ Therefore, A2B5 might be another compensate marker of CD133 to avoid GSCs isolation omission.

Several biomarkers are enriched in GSCs but rare studies adopted them for GSCs isolation. For instance, CD44 is a biomarker of MES GSCs.^19,21^ CD44 positive GSCs manifest stronger invasive ability but worse proliferative ability compared to CD133 positive GSCs.^55^ Integrin α6 co-expresses with CD133, and is associated with the ability of GSCs self-renewal and tumor initiation development.^56,57^ ALDH1 positive GSCs maintain the characteristics of GSCs including asymmetric division and sphere-formation in vitro.^58^ Nestin is expressed in both NSCs and CD133 positive GSCs.^59^ Other biomarkers like CD36,^60^ CD9,^61–63^ IL6R^64,65^ and CXCR4^66,67^ are essential to the ability of sphere-formation of CD133 positive GSCs. But there is no enough evidences to support them as a qualified hallmark in GSCs isolation.

## Proneural and mesenchymal GSCs

### Basic characteristic of PN and MES GSCs

**Fig. 2:**
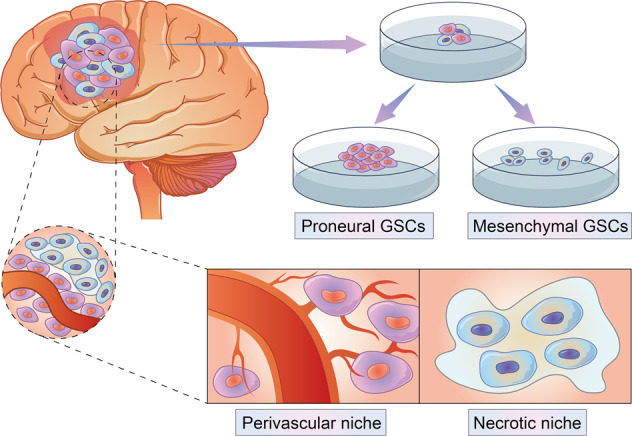
Difference between PN GSCs and MES GSCs. They manifest different growth pattern of sphere culture in vitro. PN GSCs tend to form bigger sphere and show higher growth rate than MES GSCs in vitro. In vivo, PN GSCs locate at perivascular niche while MES GSCs prefer necrotic tissue

Based on gene signatures, GSCs can be categorized as MES or PN GSCs. MES GSCs are labeled with CD44, ALDH1A3, EGFR, YKL40, IDH1-wildtype, BMI1, and GFAP whereas PN GSCs are marked with CD133, CD15, DLL3, MAP2, SOX2, OLIG2, IDH1-mutant, and EZH2.^11,19–24^ The difference in splicing profiles between PN GSCs and MES GSCs affected cell cycle, DNA repair, splicing and cilium formation.^68^ The expression profile of long non-coding RNA between PN GSCs and MES GSCs was also analyzed and prognostic related long non-coding RNAs were identified.^68^ PN GSCs prefer peri-vascular niche while MES GSCs are mainly located at the necrotic niche.^69^ PN GSCs manifest high growth rates and are able to promote tumor angiogenesis.^19^ MES GSCs show strong invasive abilities, and tumor derived from which exhibit an aggressive growth pattern.^28,70^ However, it is more difficult for MES GSCs to generate tumor than PN GSCs.^71,72^ MES GSCs have a higher resistance to radiotherapy relative to PN GSCs, and PN GSCs can be induced to transform into MES GSCs.^28,32^ The comparison between PN and MES GSCs was summarized in Table 2 and introduced with Fig. 2.

**Table 2:** The basic characteristics of PN GSCs and MES GSCs

Difference	PN GSCs	MES GSCs
Hallmarks	CD133, CD15, MAP2, SOX2, OLIG2, IDH1-mutant and EZH2	CD44, ALDH1A3, EGFR, YKL40, IDH1-wildtype, BMI1 and GFAP
Signaling pathways	The PDGF receptor-β mediated pathway; The Notch pathway; The Wnt pathway	The NF-κB pathway; FOXD-ALDH1A3 axis; glycolysis-mediated metabolism pathway
Niche	Peri-vascular niche or tumor edge tissue	Necrotic tissue
Lipid metabolism	Low	High
Glutamine utilization	Low	High
Metabolism preference	Glycolysis	Glycolysis or OXPHOS
Immunocytes infiltration	Natural killer cells	M2 macrophage; CD8^+^ cells and microglial

### The proneural-mesenchymal transition

Primary GBM tends to switch its subtype from proneural to mesenchymal at relapse and show resistance to radiochemotherapies.^11,40^ Similarly, PN GSCs can be transformed into MES GSCs during radiochemotherapies.^12,28,29,73^ The GSEA analysis was performed on differential expressed genes between MES GSCs and PN GSCs, and the result also supported that MES GSCs manifest higher resistance to tumor therapy relative to PN GSCs. Therefore, MES GSCs are able to survival from radiochemotherapies compared to PN SGCs and form therapy-resistance tumor eventually.^13^ Another study also reported, by treating GSCs with radio- or chemo-therapy, proneural related signatures (like CD133, OLIG2) in GSCs were decreased and mesenchymal-like gene signatures (like CD44, YKL40) were upregulated.^73^ Other than proneural or mesenchymal related signatures, several molecules also supported that the proneural-mesenchymal transition (PMT) in GSCs is associated with tumor resistance to tumor therapy. For instance, ZDHHC18 and ZDHHC23 are preferentially expressed in MES GSCs and PN GSCs, respectively.^74^ ZDHHC18 promotes the degradation of BMI1, an enzyme helps cells to survive under stressful environments, while ZDHHC23 assists to stabilize BMI1 expression.^74^

Differentially activated signaling pathways are also detected between PN GSCs and MES GSCs. The PDGF receptor-β mediated signaling pathway,^75^ the Notch pathway^76^ and the Wnt pathway^76,77^ are activated in PN GSCs; on the other hand, the TGF-β signaling pathway,^20^ the NF-κB signaling pathway,^19,78^ FOXD-ALDH1A3 axis^28,79^ and glycolysis-mediated metabolism pathway^28^ are activated in MES GSCs. Several pathways also were involved in the PMT and regulated cells sensitivity to therapy. The Notch signaling pathway is related to cells growth, differentiation and development. Inhibiting the activity of the Notch pathway can restore GSCs sensitivity to radiotherapy.^80^ The Wnt signaling pathway is associated with GSCs proliferation, self-renewal and tumor initiation. The dual inhibition of the Notch and Wnt pathway increased proneural related signatures in GSCs.^77^ Therefore, the Notch and Wnt pathway may be involved in the maintenance of the proneural phenotype. The activation of the NF-κB signaling pathway partly mediates the PMT in GBM.^81,82^ In GSCs, MLK4 is enriched in MES GSCs and can interact with the NF-κB signaling pathway to maintain this phenotype.^78^ On the other hand, radiotherapy increases the expression of STAT3 and C/EBP-β, the downstream of the NF-κB pathway, indicating the activation of the NF-κB pathway during the PMT.^29,83,84^ The hedgehog pathway is involved in the PMT in GBM but no research verifies its role in GSCs.^85^

In summary, molecular classification of GSCs clearly distinguishes GSCs sensitivity to radiochemotherapies. The PMT has been associated with GBM resistance to therapy, and the discovery of the PMT between GSCs subtypes might further support that theory. Therefore, the PMT can be viewed as the adaptive response of GSCs to unfavorable environment, and the inhibition of the PMT may improve patients’ overall survival outcome. Since multiple molecules or pathways related to the PMT have been identified, drugs are designed to target those mediators may improve tumor resistance to therapy.

## Metabolic phenotype of GSCs

Tumor cells metabolic reprogramming, also known as the Warburg effect, refers to tumor cells preferring glycolysis rather than the tricarboxylic acid cycle even with adequate oxygen.^86^ In contrast to GBM cells, the metabolic profile of GSCs, including lower glycolytic, lower extracellular acidification rate, less oxygen consumption and maximal respiratory capacities,^87^ is more quiescent. It should be noted that recent studies reported different metabolic phenotypes of GSCs.

One study identifies two clusters of GSCs manifesting different metabolic phenotypes, Clone A and Clone B, in the murine GBM model. Cells in Clone A are glycolysis dependent while the metabolic phenotype of Clone B can switch between mitochondrial respiration and glycolysis.^88^ One research divide GSCs into GLN-low and GLN-high GSCs based on glutamine consumption.^30^ GLN-high GSCs metabolize more glutamine to sustain its mitochondrial respiration, and the reduction of glutamine can weaken its ability in proliferation or self-renewal. Another study clusters GSCs into GSf-like GSCs and GSr-like GSCs based on metabolism profile. Cells in the former group show metabolic feature with low mobile lipids and high glutamine while cells in the latter group show the opposite.^18^ GSf-like GSCs and GSr-like GSCs also express proneural and mesenchymal related signatures, respectively. Notably, metabolic phenotype of GSCs corresponds with molecular classification. GSf-like GSCs express proneural related signatures while mesenchymal related signatures are enriched in GSr-like GSCs. Another study reported that GSCs with activated lipid metabolism and reduced glucose consumption are resistance to radiochemotherapies.^89^ Activated glutamine metabolism is also associated with GSCs resistance to radiotherapy.^26^ Therefore, therapy-resistant GSCs consume less glucose, with activated glutamine and lipid metabolism by contrasting with therapy sensitive GSCs.

In summary, there are two metabolic phenotypes of GSCs. The first phenotype is GSCs dependent on aerobic glycolysis. This type of GSCs metabolizes glucose to supply cells proliferation. The other phenotype is more complicated. GSCs can switch between glycolysis and OXPHOS according to extracellular stimulation. Instead of consuming glucose, GSCs in this type prefer to metabolize glutamine to initiate OXPHOS. Glycolysis is only activated when the supply of glutamine is insufficient.^30^ Besides, this type of GSCs contains more mobile lipids in cytoplasm, which indicates the activated lipid metabolism-related pathways. As aforementioned, metabolic phenotype of GSCs affects its sensitivity to cancer therapy. More effort on exploration about the mechanisms of how abnormal metabolic pattern affects cells resistance to therapy remains to be urgently needed.

## Biological behavior classification

Classification based on GSCs biological behavior classifies GSCs into qGSCs or pGSCs.^25,27^ ‘Quiescent cells’ refers to cells with slow cell cycle relative to normal cells, and cells are able to quit this state when necessary.^33,90,91^ Label-retaining assay is able to distinguish quiescent GSCs from tumor.^27,92^ Apart from that, recent studies reported that isolation of qGSCs by marking the promoter of nuclear receptor tailless of GSCs with GFP^93^ or based on GSCs’ sensitivity to a different chemical compound.^94^ In 3D organoids culturing system, stem-like cells at the periphery showed strong proliferative ability while cells in the hypoxic core more quiescence.^39^ However, the accuracy of qGSCs isolation between those protocols is lack of comparison.

The proliferation ratio of pGSCs is significantly quicker than qGSCs but there are no specific molecular hallmarks to distinguish them.^25,33^ Differentially expressed genes profile identifies SAT1 and ID1 upregulated in qGSCs while EGFR enriched in pGSCs.^95^ pGSCs are mostly located in the perivascular niche, which is similar to PN GSCs, while qGSCs are located in necrotic niche.^33,96^ BMP and TGF-β signaling pathways are selectively activated in qGSCs and pGSCs respectively.^95^ Besides, biofunction prediction suggests that dysregulated genes in qGSCs are related to tumor immune landscape and tumor resistance to therapy, while genes in pGSCs are associated with cell proliferation.^95^

### Potential regulators of biological behavior of GSCs

The expression profile of cell cycle-related genes reveals the mechanism of different biological behavior of GSCs. For instance, cyclin B1, CDKN1A and G0S2 expression are dysregulated in qGSCs.^33^ Accumulation of p27 at G0 phase in qGSCs is associated with the maintenance of cells quiescence.^97^ Factors like Ca2+ influx related genes expression (like CACNB1, CAPS, CACNA2D1, PKD2 and ORAI2),^98^ the activity of Notch signaling pathway,^99^ mitochondrial shape^96^ and hypoxia and acidic niche^39^ are also raised for their role in quiescence state.

Other potential regulators involved in the biological behavior of GSCs are also summarized. DOCK4 and β-catenin affect GSCs proliferative ability through influencing GSK3-β activity.^100^ NGF and its receptors control GSCs proliferation.^101^ The proliferation rate of GSCs can be inhibited by silencing the expression of STAT3 and integrin α6.^102,103^ Those regulators can affect the proliferation ability of GSCs but their role in pGSCs or qGSCs remain elusive.

### Biological behavior transition

The proliferation-quiescence transition is termed as pGSCs entering the ‘quiescence’ status. This transition can be induced by hypoxia or an acidic environment through altering mitochondrial shape and cytometric calcium concentration of GSCs.^33^ Notably, the ratio of qGSCs in tumor is positively correlated with tumor recurrence times.^33^ The population of qGSCs is increased after treating GBM with RTK inhibitors, and the activity of the Notch pathway and KDM expression is also increased.^104^ In general, pGSCs can be transformed to qGSCs under the stimulation of unfavorable environment or radiochemotherapies, and this transition could be a novel mechanism of tumor resistance to therapy.^25,92,93,105^

Since the ‘quiescence’ state is a reversible state, pGSCs can also generate from qGSCs.^93^ The GINS complex (comprise of SLD5, PSF1, PSF2, and PSF3) re-initiates cell cycle in qGSCs by altering cell-cycle-related genes expression.^106^ Evidence supporting the quiescence-proliferation transition is insufficient, and this transition might be related to tumor recurrence.

Biological behavior of GSCs also sheds light on the mechanism of GSCs sensitivity to therapy. For instance, qGSCs can survive from an unfavorable environment and develop tumor by quitting the quiescence state. pGSCs can enter the quiescence state when the environment is not favorable for survival. This dual-transition highlights the mechanism of tumor recurrence and tumor resistance to therapy.

## Internal connection between different classifications of GSCs

Previous studies subdivided GSCs into PN GSCs or MES GSCs based on molecular classification. Notably, two metabolic phenotypes of GSCs are also associated with PN GSCs or MES GSCs. According to the metabolic profile and molecule signatures, cells in Clone A, GSf-like GSCs and GLN-low GSCs are PN GSCs while cells in another group (Clone B, GSr-like GSCs and GLN-high GSCs) are MES GSCs. Therefore, PN GSCs depend on aerobic glycolysis while the metabolic phenotype of MES GSCs is more flexible.^26^

MES GSCs consume glutamine and glucose to supply OXPHOS and glycolysis, respectively. Since glutamine can replenish lipid biosynthesis precursors and supply mitochondrial respiration,^26^ glutamine and lipid metabolism-related pathways are also activated in MES GSCs.^18^ Multiple studies supported that activated glutamine and lipid metabolism are involved in tumor resistance to therapy.^31,107,108^ Thus, the mechanism of MES GSCs shows resistance to therapy might relate to this metabolic phenotype.

On the other hand, PN GSCs and MES GSCs manifest stronger ability in proliferation and migration, respectively. The connection between molecular classification and GSCs biological behavior is unclear. Given the slow cell cycle of qGSCs, pGSCs might be a group of cells containing PN GSCs and MES GSCs simultaneously. However, pGSCs and PN GSCs both are located at perivascular niche while qGSCs and MES GSCs prefer necrotic niche.

**Fig. 3:**
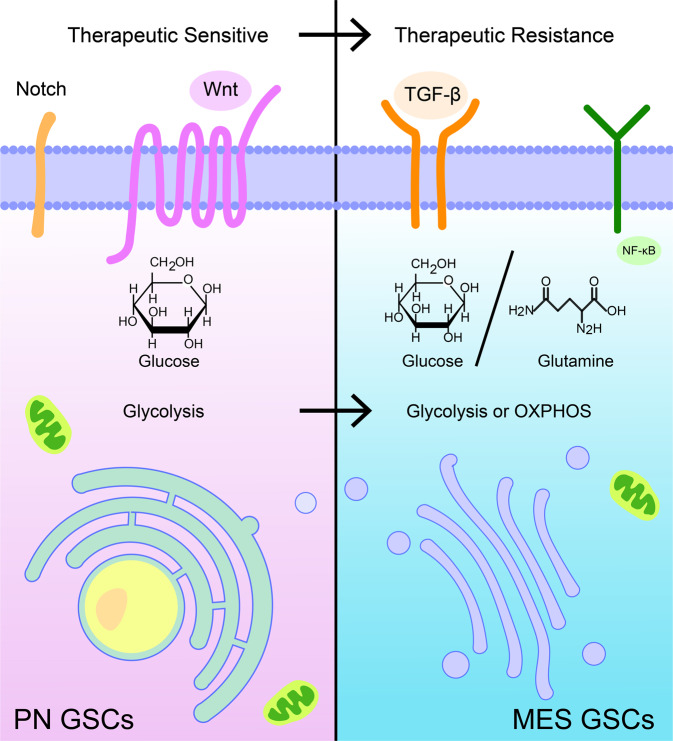
The adaptive transition within GSCs classifications. Therapeutic sensitive GSCs like PN GSCs can transform into MES GSCs which show resistance to cancer treatment. PN GSCs mostly dependent on glycolysis, and MES GSCs switch its metabolism between glycolysis and OXPHOS. PN GSCs metabolize glucose while MES GSCs can switch between glucose and glutamine. The Notch and Wnt pathway are preferentially activated in PN GSCs; the activation of TGF-β pathway and NF-κB pathway are mostly observed in MES GSCs

Three classifications, molecular, biological behavior, and metabolic phenotype all elaborate only one feature of GSCs. Within each group, GSCs can also be grouped as therapy sensitive or resistant cells. Transition restricted to each classification clearly map the response of GSCs to therapy or unfavorable environment. Several studies have confirmed the connection between molecular classification and metabolic phenotype. However, their association with GSCs biological behavior is unclear. Figuring out the internal connection between different classifications can reveal the feature of therapy-resistant GSCs and promote clinical management (Fig. 3).

### The relationship between NSCs and GSCs

Subventricular NSCs (also called as astrocyte-like NSCs or type B cells) contain two groups of cells, B1 astrocytes and B2 astrocytes. B2 astrocytes are non-neurogenic astrocytes. B1 astrocytes asymmetrically split into type C cells (also known as transit-amplifying progenitor cells) which will differentiate into type A cells (also known as neuroblasts) or oligodendrocyte precursor cells in the end. In the meantime, B1 astrocytes can be subdivided into quiescent NSCs (qNSCs) and active NSCs (aNSCs) based on their biological behavior. Type A cells can form neurons, and oligodendrocyte precursor cells differentiate into oligodendrocytes or astrocytes.^109,110^

Several studies reported that GSCs are derived from subventricular NSCs,^111–113^ and the fact that by engineering p53,^114^ EGFR^115^ or H-Ras^V12 88^ in NSCs can induce the formation of GSCs. Gnomically, NSCs and GSCs share common gene signatures including SOX2, NESTIN, OLIG2, CD133, YKL40, et al.^116,117^ CD133 and Nestin are both expressed in B1 astrocytes and PN GSCs; EGFR is mainly enriched in type C cells and MES GSCs^118,119^ (Table 3). This hallmark similarity implies the association between NSCs and GSCs.

**Table 3:** Common gene signatures of NSCs

Cells	GFAP	Nestin	CD133	EGFR	CD15
qNSCs	+	−	+	−	+
aNSCs	+	+	+	+	+
B2 astrocytes	+	+	−	+	−
Type C cells	−	+	−	+	+
Type A cells	−	−	−	−	−
PN GSCs	−	+	+	−	+
MES GSCs	−	−	−	+	+

Metabolically, NSCs depend on glycolysis to maintain its stemness, but its differentiation is involved in the activation of several metabolic pathways including elevated fatty acid consumption, increased lipogenesis, decreased glycolysis and activated OXPHOS.^120–122^ This metabolic phenotype transition during NSCs differentiation is similar to the PMT. Glycolysis dependent type C cells show tolerance to the hypoxia environment,^123^ but its proliferation still relies on absorbing extracellular fatty acid and activating de novo lipogenesis.^124^ The metabolic patterns transition during the differentiation of NSCs is similar to GSCs adaptive transition.

The biological behavior of qNSCs and aNSCs is similar to that of qGSCs and pGSCs, respectively.^125,126^ Besides, aNSCs and pGSCs both show sensitivity to temozolomide but qNSCs and qGSCs can survive from it.^127^ Therefore, the nature of NSCs may also affect GSCs sensitivity to therapy.

Another study pioneered exploring the association between GSCs and NSCs by performing single-cell RNA sequencing analysis.^128^ They proved that the apex of GBM hierarchy is progenitor cancer cells, and most of them carry with proneural signature while few of them are classified as mesenchymal or classical. They also identify an un classified type of GSCs which show similarity with progenitor cancer cells.

**Fig. 4:**
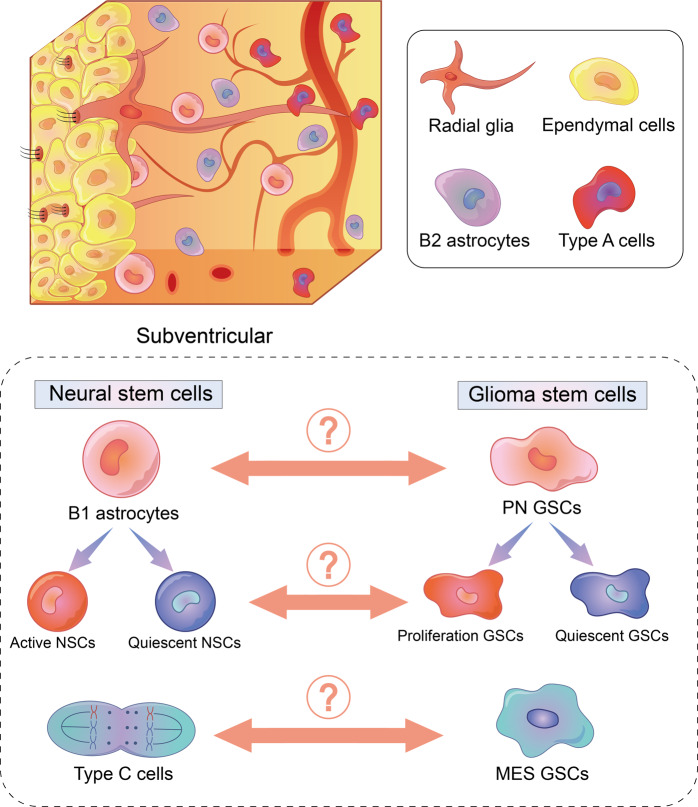
Association between subventricular NSCs and GSCs. Similarities, including genomic characteristics and metabolism pattern, are found between PN GSCs and B1 astrocytes, MES GSCs and type C cells. The biological behavior of B1 astrocytes is similar to GSCs. However, the explicit relationship between NSCs and GSCs remains unknown

Taken together, similarity in transcriptomic signature, metabolic profile, biological behavior and single-cell RNA sequencing analysis highlighted the internal correlation of GSCs and NSCs. Furthermore, it may be hypothesized that GSCs are derived from B1 astrocytes, and the PMT is the glioma version of the B1 astrocytes differentiation. Nevertheless, evidences from some studies make different voice. For instance, CD44, hallmarks of MES GSCs, is expressed on astrocyte-restricted precursors that do not express on NSCs. Metabolic profile of PN GSCs is similar to type C cells instead of B1 astrocyte.^88^ The origin of IDH wildtype glioma and IDH mutant glioma might different.^129^ Since differentiation of NSCs is a complicated, precise, dynamic process, their internal relationship with GSCs still needs more investigation (Fig. 4).

## Niche and GSCs

Niche is a special microenvironment where stem cells are preserved. The niche of GSCs comprises of multiple components including endothelial cells (ECs), arterioles, immunocytes, fibroblasts, NSCs, pericytes, et al.^130^ Several studies reported five types niches in GBM, including peri-vascular niches,^131^ peri-arteriolar niches,^132^ peri-hypoxic niches,^133^ peri-immune niches^134^, and ECM niches,^135^ based on their unique traits. Those niches share similar features and interrelate with each other. Therefore, a comprehensive model integrating those niches called hypoxic peri-arteriolar niche was proposed.^136^ More importantly, this dynamic model simplifies the catalog of GSCs niches and improves the understanding of the interconnection between niches and GSCs. However, discussion about the relationship between MES GSCs and this model is not mentioned.

### Blood vessels and vasculogenic mimicry

Blood vessels distribution in GBM varies from normal brain tissue. ECs, pericytes and smooth muscle cells are constituents of blood vessels, and they both affect GSCs. For instance, ECs are associated with the maintenance of GSCs stemness by activating the Notch pathway.^137,138^ On the other hand, GSCs can transdifferentiate into ECs, pericytes and smooth muscle cells, and involve in the formation of vasculogenic mimicry.^139–141^ GSCs-derived pericytes contribute to tumor resistance to therapy by altering the permeability of the blood-brain barrier.^142–144^ Besides, several molecules are involved in this process, including Flk-1,^145^ CDH5,^146^ YKL40,^147^ KDR^148^, and VEGF.^149^ In summary, GSCs are closely associated with tumorigenesis and vasculogenic mimicry.

### Hypoxia and acidic tumor microenvironment

Hypoxia and acidic are critical characteristics of tumor microenvironment.^150,151^ The survival probability of PN in hypoxia and acid environment is lower than MES GSCs.^74^ HIF-2α is involve in maintaining the stemness of GSCs and contributes to the PMT.^55^ Hypoxia can activate glutamine metabolism-related pathway in tumor cells.^152,153^ Besides, hypoxia and acidic tumor microenvironment affect the proliferation-quiescence transition of GSCs.^33^ Together, those results indicate that niche is involved in GSCs resistance to therapy.

### Immunocytes infiltration

Immunocytes infiltration of PN and MES GSCs is different. For instance, qGSCs upregulate the expression of T cell targeted antigen and are infiltrated with more T cells than pGSCs.^154^ MES GSCs have higher infiltration of CD8 positive T cells and microglial than PN GSCs.^155^ Tumor-associated macrophages are derived from bone marrow-derived monocytes, microglial cells and GSCs.^156^ PN GSCs induce the formation of tumor-associated macrophages and recruit M2 tumor-associated macrophages.^36,157^

Compared with MES GSCs, PN GSCs increase the expression of MHC I, CD40 and CD86 and downregulate the expression of MHC II and CD80. B7-H1, an inhibitory molecule of T cells, is also increased in PN GSCs.^158^ Hypoxia microenvironment promotes PN GSCs to release immunosuppressive cytokines.^159^ PN GSCs show resistance to TGF-β stimulation and its low TGF-β expression indicates that TGF-β acts an immunosuppressive role in PN GSCs.^155^ Together, those results reveal lower immunocytes infiltration in PN GSCs than MES GSCs, and this difference might relate to the efficacy of GSCs sensitivity to immunotherapy.

The comprehensive model of niche allows a clearer view of the relationship between niche and GSCs. The components of the niche are complicated and dynamic. GSCs affect the formation of niche, in turn, niche components like pericytes or characteristics like hypoxia influence the subtype of GSCs as well as GSCs sensitivity to therapy. Currently, whether niche contributes to GSCs resistance to therapy is still unclear.

## Implications on treatments

**Fig. 5:**
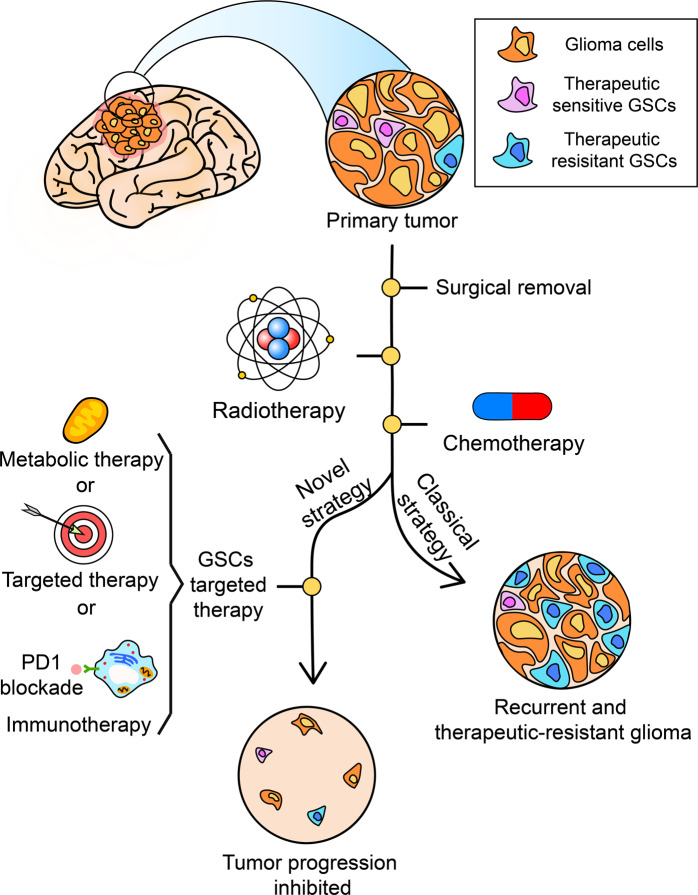
Therapy resistant GSCs like MES GSCs can survive from a classical strategy like chemo- or radio-therapy. In the meantime, the combination of GSCs targeted therapy and classical strategy may improve treatment efficiency

As aforementioned, a different subtype of GSCs carry with different characteristics and show different sensitivity to tumor therapy. Therefore, targeting to GSCs selectively may be an option. In recent years, progresses in selectively targeting to GSCs subtype have been reported, and the section summarized those progresses (Fig. 5).

### Radiotherapy

PN GSCs can transform into MES GSCs by treating with radiotherapy and show resistance to radiotherapy.^28^ Multiple studies revealed that interfering the PMT related pathway can restore GBM sensitivity to radiotherapy.^160–164^ However, the efficacy of inhibiting the PMT in GSCs is not verified. The inhibition of relevant metabolic pathways in GSCs might also be a novel treatment to restore GSCs sensitivity to radiotherapy.

### Chemotherapy

The relationship between GSCs and chemotherapy is more complicated. Temozolomide is the most common and efficient chemotherapeutic agent in clinical application to treat GBM. In primary GBM, PN GSCs are resistant to multiple chemotherapeutic agents including temozolomide.^165,166^ Another study reported temozolomide can inhibit GSCs proliferation with still a small group of GSCs survived.^167^ Besides, the expression of MGMT, a biomarker to predict GBM sensitivity to temozolomide, can also predict GSCs sensitivity to temozolomide regardless of molecular signatures.^167^ Notably, the combination of temozolomide and perillyl alcohol has a lethal effect on PN GSCs derived and MES GSC derived GBM.^168^ On the contrary, MES GSCs derived GBM show resistance to temozolomide and gradually lost its mesenchymal related signatures during treating with temozolomide.^32^ In the meantime, tumor sensitivity to radiotherapy is restored.^32^ In general, MES GSCs have a higher expression of several therapy resistance-related genes compared to PN GSCs. But their sensitivity to chemotherapy do not show no significant difference, and MGMT is still a qualified biomarker to predict GSCs sensitivity to temozolomide.

### Metabolic therapy

Activated glutamine metabolism in MES GSCs is associated with GSCs resistance to radiochemotherapies and GSCs proliferation.^89^ EGCG, an inhibitor of transglutaminase, can restore GSCs sensitivity to temozolomide and inhibit GSCs proliferation.^30,169^ Dichloroacetate, the inhibitor of pyruvate dehydrogenase kinase inhibitor, can increase GSCs sensitivity to radiotherapy.^170^ In MES GSCs, glutamine serves as a metabolic substrate of OXPHOS, and pyruvate dehydrogenase kinase is also critical to supply OXPHOS. Therefore, the activation of mitochondrial in MES GSCs might be connected with its resistance to therapy. Metformin, an inhibitor of mitochondrial complex I, can affect tumor cells resistance to therapy but its role in GSCs is not confirmed.^171^

### Immunotherapy

Immunotherapy targeting GSCs or adopting GSCs as therapeutic methods to treat GBM has made some progress in recent years.^172,173^ Besides, immune check point genes and antigen presentation genes are differentially expressed on PN GSCs and MES GSCs as aforementioned.^158^ Immunocytes infiltration difference is identified in molecular classification and biological behavior classification.^154,155^ Metabolic phenotypes also affect GSCs sensitivity to immunotherapy. For instance, ‘metabolic check point’like glucose depletion and hypoxia affect the function of tumor infiltrated immunocytes.^174^ Together, those results suggest that the response of GSCs to immunotherapy might also differ from each other.^154,175^ However, the association between GSCs and immunotherapy is not clear. Notably, a recent study reported tumor immune escape can be inhibited by blocking glutamine metabolism-related pathways, indicating that a similar strategy could be applied to GSCs.^176^

### Other treatments

Anti-VEGF therapy is a novel strategy to treat cancer but its efficacy in GBM is not optimistic.^3,177^ Bevacizumab, VEGF inhibitor, promotes vasculogenic mimicry formation by PN GSCs^148^ which might be associated with its failure of treating primary GBM.^178^

Tumor-treating fields inhibit tumor cells proliferation to prevent GBM progression. It can also be applied to treat tumor derived from GSCs.^179–182^ However, the association between GSCs resistance and tumor-treating fields is not clear.

The previous study reported GSCs to respond differently to targeted therapy.^183^ Since various strategies such as nanoparticles^184,185^ and Zika virus^186–188^ can be applied to target GSCs, it is critical to treat GSCs with combined therapeutic options to improve treatment efficacy. A recent study summarized the efficacy of strategy that by targeting SOX2 on stem-like cells can inhibit tumor progression.^189^

### Clinical trials targeting GSCs

Apart from traditional treatments of GBM, several clinical trials proposed a novel strategy in recent years. All information about clinical trials is obtained from public clinical trial databases (https://clinicaltrials.gov/). Seventy-eight results in total are obtained by setting ‘glioma stem cells’ as the keyword, and nineteen of them are about targeting GSCs or adopting NSCs as therapeutic means (Table 4). Two out of six completed clinical trials have published their results. The efficacy of therapy adopting NSCs as therapeutic means can be evaluated by MRI.^190^ Dendritic cells loaded with GSCs-derived mRNA can inhibit GBM growth.^191^ Only one trial which used GSCs as the antigen source of the vaccine was terminated due to limit efficacy and extreme toxicity (ClinicalTrial.gov Identifier: NCT01400672). However, few clinical trials take the difference in GSCs into account.

**Table 4:** Clinical trials target on GSCs or adopt NSCs as therapeutic means

NCT number	Title	Status	Target or medium	Therapy
NCT02039778	Stem cell radiotherapy and temozolomide for newly diagnosed high-grade glioma	Terminated	GSCs	Radio- and chemo-therapy
NCT03072134	Neural stem cell-based virotherapy of newly diagnosed malignant glioma	Completed	NSCs	Virotherapy
NCT01872221	Study of the capacity of the MRI spectroscopy to define the tumor area enriched in glioblastoma stem cells. Proof of concept study	Completed	GSCs	Radio- and chemo-therapy
NCT02192359	Carboxylesterase-expressing allogeneic neural stem cells and irinotecan hydrochloride in treating patients with recurrent high-grade gliomas	Recruiting	NSCs	Genetically modified therapy
NCT01172964	A pilot feasibility study of oral 5-fluorocytosine and genetically modified neural stem cells expressing *E. Coli* cytosine deaminase for treatment of recurrent high-grade gliomas	Completed	NSCs	Genetically modified therapy
NCT02010606	Phase I study of a dendritic cell vaccine for patients with either newly diagnosed or recurrent glioblastoma	Active, not recruiting	GSCs	Immunotherapy
NCT02055196	Genetically modified stem cells and irinotecan hydrochloride in treating patients with recurrent high-grade gliomas	Withdrawn	NSCs	Genetically modified therapy
NCT02015819	Genetically modified neural stem cells, flucytosine, and leucovorin for treating patients with recurrent high-grade gliomas	Active, not recruiting	NSCs	Genetically modified therapy
NCT01171469	Vaccination with dendritic cells loaded with brain tumor stem cells for progressive malignant brain tumor	Completed	GSCs	Immunotherapy
NCT03956706	Study of stereotactic radiosurgery to the subventricular zone in malignant gliomas	Recruiting	NSCs	Radiotherapy
NCT01567202	Study of DC vaccination against glioblastoma	Recruiting	GSCs	Immunotherapy
NCT00473408	The effect of radiotherapy and temozolomide on the tumor vasculature and stem cells in human high-grade astrocytomas	Terminated	GSCs	Radio- and chemo-therapy
NCT03632135	Standard chemotherapy vs. chemotherapy guided by cancer stem cell test in recurrent glioblastoma	Recruiting	GSCs	Chemotherapy
NCT02654964	Cancer stem cell high-throughput drug screening study	Unknown status	GSCs	Chemotherapy
NCT03548571	Dendritic cell immunotherapy against cancer stem cells in glioblastoma patients receiving standard therapy	Recruiting	GSCs	Immunotherapy
NCT00846456	Safe study of dendritic cell (DC) based therapy targeting tumor stem cells in glioblastoma	Completed	GSCs	Immunotherapy
NCT00890032	Vaccine therapy in treating patients undergoing surgery for recurrent glioblastoma multiforme	Completed	GSCs	Immunotherapy
NCT01400672	Imiquimod/brain tumor initiating cell (BTIC) vaccine in brain stem glioma	Terminated	GSCs	Radio- and immune-therapy
NCT02177578	Subventricular zone (SVZ) and temozolomide in glioblastoma multiforme	Recruiting	NSCs	Radio- and chemo-therapy

## Conclusion and prospection

In this review, different classifications of GSCs are summarized and integrated. However, there are several questions about GSCs classification. First, several genes signatures of the proneural or mesenchymal subtype are identified nowadays, but few of them can be applied to GSCs isolation. Considering the PMT in GSCs, a precise method to isolate GSCs can bring about a more accurate result. Second, the inner relationship between biological behavior classification and the other two classifications is not clear. pGSCs seem to be connected with PN GSCs but no similarity is found between qGSCs and other subtypes of GSCs. Third, the metabolic phenotype of GSCs requires more attention. One study which subdivided GSCs into three groups (Cluster1a, Cluster1b and Cluster2) reported that the molecular signatures of Cluster1a are similar to those of PN GSCs whereas cells in the other two groups are similar to those in MES GSCs.^192^ Notably, Cluster1 (including Cluster1a and Cluster1b) manifests a flexible metabolic phenotype while Cluster2 mainly depends on glycolysis. Obviously, this conclusion is non consistent with previous results.^18,30,72,88^ Each classification mentioned above merely reveals one feature of GSCs. An integrative analysis of those classifications will provide a better understanding of GSCs.

Multiple studies proved tumor cell adaptive survival from anti-tumor therapy, and this process was viewed as tumor therapeutic response^193^ Therapy sensitive or resistant GSCs are also identified in each classification. For instance, MES GSCs, glutamine dependent GSCs and qGSCs show nature resistance to cancer therapy. Transition restricted to each classification like the PMT and the proliferative-quiescent transition is highly associated with GSCs resistance to cancer therapy. Besides, MES GSCs can transform their metabolic pattern according to the context, indicating that MES GSCs are hard to be affected by constraining its nutrition supply. All kinds of GSCs adaptive transition not only reveal the mechanisms of tumor recurrence and tumor resistance to cancer therapy but also highlight multiple potential targets for future research. Therefore, molecular signatures, pathways or metabolic pattern involved in GSCs adaptive transition can be served as potential targets to improve therapeutic efficacy.

The inner relationship between NSCs and GSCs is not clear. Multiple studies suggested that NSCs are the derivation of GSCs, in the meantime, other studies further confirmed the similarity between different GSCs subtypes and NSCs differentiation lineage. Therefore, GSCs adaptive transition might share common features with NSCs differentiation lineage. Niche is another factor that affecting GSCs adaptive transition, and GSCs influence the formation of the niche in turn. Besides, immunocytes infiltration and vasculogenic mimicry can affect tumor response to cancer therapy. Therefore, NSCs, niche and GSCs interact with each other. But more in-depth mechanisms remain to be revealed. In general, GSCs is a novel breakpoint for understanding tumor recurrence and tumor resistance to cancer therapy.
